# Systematic analysis of bacterial lipopolysaccharide-related genes and immune cell infiltration characteristics in pediatric septic shock using integrated bioinformatics and machine learning approaches

**DOI:** 10.3389/fbinf.2026.1729982

**Published:** 2026-05-29

**Authors:** Junying Qiao, Lanlan Zou, Jianchuang Zhao, Chenhang Cui, Xianjie Huang, Yazhen Fan

**Affiliations:** Department of Pediatric Critical Care Medicine, The Third Affiliated Hospital of Zhengzhou University, Zhengzhou University, Zhengzhou, China

**Keywords:** bioinformatics, biomarkers, immune infiltration, lipopolysaccharide-associated genes, machine learning, pediatric septic shock

## Abstract

**Background:**

Bacterial lipopolysaccharide (LPS) play a crucial role in triggering dysregulated immune responses in pediatric septic shock, profoundly influencing disease onset and progression. This study systematically investigated the differential expression of LPS-related genes, constructed diagnostic models, explored regulatory networks, analyzed immune cell infiltration, and identified potential therapeutic targets of traditional Chinese medicine for pediatric septic shock.

**Methods:**

Three publicly available pediatric septic shock datasets (GSE26440, GSE9692, and GSE13904) were retrieved from gene expression repositories. Differentially expressed genes (DEGs) were identified, and key module genes were determined using weighted gene co-expression network analysis (WGCNA). These genes were intersected with LPS-related genes curated from genomic databases. To improve biomarker screening precision, the Least Absolute Shrinkage and Selection Operator (LASSO) regression and Support Vector Machine–Recursive Feature Elimination (SVM-RFE) algorithms were applied to identify feature genes and construct a diagnostic model, which was validated using an independent dataset. Functional enrichment analyses were performed based on diagnostic model-derived scores. Immune cell infiltration was quantified using the ssGSEA algorithm, and transcription factor (TF)–gene regulatory networks were constructed to elucidate underlying molecular mechanisms. Moreover, drugs relevant to pediatric sepsis over the past decade were extracted from the COREMINE database, and their bioactive components underwent molecular docking with the identified feature genes to evaluate binding affinity.

**Results:**

Four LPS-related feature genes—IL10, MMP9, S100A12, and STAT3—were identified as potential diagnostic biomarkers of pediatric septic shock. The diagnostic model built using the Stepwise Generalized Linear Model (StepGLM [backward]) combined with LASSO achieved excellent performance, with an average area under the ROC curve (AUC) of 0.994. Enrichment analyses revealed that high-score samples were significantly associated with inflammatory and immune hyperactivation pathways, whereas low-score samples were enriched in homeostatic or protective pathways. Immune infiltration analysis revealed marked differences among multiple immune cell types, including lymphocyte subsets, neutrophils, and macrophages. Notably, MMP9 expression showed a strong positive correlation with activated dendritic cells. Protein–protein interaction and regulatory analyses revealed a TF–gene network comprising 26 nodes and 32 edges and a miRNA–gene network with 71 nodes and 67 edges. Furthermore, Chrysanthemum indicum was identified as a promising therapeutic candidate, with luteolin and quercetin as its principal active ingredients. Molecular docking analyses confirmed stable binding affinities between these compounds and the key feature genes.

**Conclusion:**

This integrative bioinformatics and machine learning study identified IL10, MMP9, S100A12, and STAT3 as LPS-associated signature genes in pediatric septic shock. These genes are intricately involved in immune dysregulation and may serve as potential diagnostic biomarkers and therapeutic targets. The findings provide novel insights into the molecular mechanisms and treatment strategies for pediatric sepsis.

## 1 Introduction

Sepsis is a life-threatening condition characterized by organ dysfunction and impairment resulting from a dysregulated host response to infection (Cajander et al., 2024). In 2017, an estimated 25 million children worldwide developed sepsis, resulting in more than three million deaths (Rudd et al., 2020). Septic shock represents the most severe manifestation of sepsis and is a leading direct cause of mortality. Clinically, it is defined by the presence of at least one cardiovascular criterion from the Phoenix score—namely, age-adjusted severe hypotension, blood lactate levels exceeding 5 mmol/L, or the requirement for vasopressor therapy (Sanchez-Pinto et al., 2024). Even in high-resource healthcare settings, the in-hospital mortality rate for pediatric septic shock remains approximately 10.8% (Schlapbach et al., 2024). Therefore, early identification and precise management of pediatric septic shock are essential to reducing mortality and improving clinical outcomes.

Bacterial lipopolysaccharide (LPS), also known as endotoxin, is a pivotal initiator of the inflammatory cascade in sepsis and septic shock. It provokes the systemic release of dysregulated and potentially lethal inflammatory mediators and procoagulant factors (Dm and Ja, 2023). Structurally, LPS comprises three components: lipid A, a core polysaccharide, and the O-antigen (Wang and Quinn, 2010). Functionally, LPS is recognized as a prototypical pathogen-associated molecular pattern (PAMP) that triggers sepsis and its shock phase. When Gram-negative bacteria undergo lysis or release outer membrane vesicles, LPS enters the circulation and is recognized by the host immune system, rapidly inducing an intense inflammatory response and vascular endothelial dysfunction (Wang et al., 2023). The immune recognition of LPS is highly complex, involving multiple signaling levels and pathways. The canonical pathway is mediated through Toll-like receptor 4 (TLR4) signaling. Circulating LPS first binds to LPS-binding protein (LBP) and is subsequently transferred to TLR4 via CD14 (Yamamoto and Akira, 2010), The resulting LPS/MD2/TLR4 complex activates two major downstream signaling axes—MyD88/NF-κB and TRIF/IRF3—leading to the production of proinflammatory cytokines such as TNF-α, IL-6, and IFN-β (Lu et al., 2008). Despite extensive mechanistic studies, a systematic investigation of the regulatory networks and immune cell infiltration patterns associated with LPS-related genes in pediatric septic shock has yet to be conducted.

The essence of septic shock lies in the dysregulation of the host’s response to infection, leading to life-threatening organ dysfunction (Singer et al., 2016). As a potent immune activator, LPS induces a dynamic imbalance in the immune system through complex mechanisms (Mazgaeen and Gurung, 2020). The early inflammatory response triggered by LPS, characterized by a “cytokine storm,” represents a pivotal pathophysiological change in the early stages of septic shock. Upon binding to TLR4, LPS activates signaling pathways such as NF-κB and MAPK, prompting immune cells like monocytes and macrophages to massively release proinflammatory mediators (Park and Lee, 2013). Additionally, neutrophils and dendritic cells exhibit high activation during the early inflammatory phase, collectively forming the immune-inflammatory landscape of early sepsis (Biswas and Lopez-Collazo, 2009). In stark contrast to this early hyperinflammatory response, advanced sepsis shock often manifests as an immunoparalytic state characterized by suppressed immune cell function, increased lymphocyte apoptosis, and heightened susceptibility to secondary infections (Hotchkiss et al., 2013). Thus, the immune imbalance associated with bacterial lipopolysaccharide-induced septic shock encompasses both the early “inflammatory storm” and the subsequent immunosuppression. These two phases are intertwined, jointly determining the pathological progression and clinical outcomes of sepsis (Van Der Poll et al., 2021).

Given the pivotal role of bacterial lipopolysaccharide–associated genes in the immune dysregulation underlying pediatric septic shock, this study aimed to comprehensively identify LPS-related signature genes using integrated bioinformatics and machine learning approaches. Functional enrichment and regulatory network analyses were performed to elucidate the biological pathways and molecular interactions associated with these genes. In addition, diagnostic models were constructed to assess their predictive value, and correlations between gene expression and immune cell infiltration were systematically evaluated. Furthermore, potential therapeutic drugs and their key bioactive compounds were screened, and molecular docking analyses were conducted to evaluate their binding affinities with the identified signature genes.

## 2 Methods

### 2.1 Acquisition and screening of expression profile data

The GSE26440 dataset was obtained from the Gene Expression Omnibus (GEO, https://www.ncbi.nlm.nih.gov/geo/) database (Barrett et al., 2005) using the keywords “pediatric septic shock” and “*Homo sapiens*” as the training set. The GSE9692 and GSE13904 datasets were used as independent validation sets. Specifically, GSE26440 contained 98 pediatric septic shock samples and 32 healthy controls; GSE9692 included 30 pediatric septic shock samples and 15 healthy controls; and GSE13904 comprised 106 pediatric septic shock samples and 18 healthy controls. All samples were categorized into disease and control groups. We employed microarray-based sequencing using the GPL570 platform. The processed probe expression matrix was directly downloaded, along with the corresponding platform annotation file, which was used to convert probe IDs into gene symbols. When multiple probes corresponded to the same gene symbol, they were consolidated accordingly. Furthermore, the Comparative Toxicogenomics Database (CTD, http://ctdbase.org/) (Davis et al., 2023) was used to identify genes associated with lipopolysaccharide.

### 2.2 Identification of differentially expressed genes

Differentially expressed genes (DEGs) between disease and control groups were identified using the limma package (Version 3.6.2) (Liu et al., 2021) in R software. Genes with an adjusted p-value <0.05 and |log_2_FC| > 0.5 were considered significantly differentially expressed. Volcano plots were generated using the ggplot2 package (Version 3.5.1) (Gustavsson et al., 2022) and the top ten upregulated and downregulated genes, ranked by fold change, were visualized as heatmaps using the ComplexHeatmap package (Version 2.20.0) (Gu, 2022).

### 2.3 Weighted gene Co-expression network construction

Weighted gene co-expression network analysis (WGCNA) (Langfelder and Horvath, 2008) is a systems biology approach widely applied in studies of disease and trait association. For the weighted gene co-expression network analysis (Version 1.73), disease and control groups from the GSE26440 dataset were used as phenotypic traits. The expression matrix was filtered by selecting the top 5,000 genes based on the absolute median deviation (MAD), which served as the input for constructing the co-expression network. WGCNA was then performed to identify modules associated with pediatric septic shock. Initially, sample clustering was performed to identify and remove outliers. The soft-thresholding power (abline h = 0.85) was determined using the pickSoftThreshold function to achieve scale-free topology. A gene adjacency matrix was constructed and subsequently transformed into a topological overlap matrix (TOM). Modules were identified using dynamic tree cutting, and module–trait correlations were calculated. Modules with |R| > 0.5 and *p* < 0.05 were defined as key modules, and their genes were considered disease-associated. The intersection between DEGs, WGCNA-derived genes, and lipopolysaccharide-related genes was determined and visualized using the VennDiagram package (Version 1.7.3) (Chen and Boutros, 2011).

### 2.4 Construction of the protein–protein interaction network

Protein–protein interaction (PPIs) are essential for numerous biological processes, including signal transduction, gene regulation, metabolism, and cell cycle control (Jiang et al., 2023). In this study, an intersection gene protein–protein interaction (PPI) network was constructed using the STRING database (Szklarczyk et al., 2022). The genes used were intersection genes selected from the Venn diagram, and the species was set as human (*H. sapiens*). The confidence score threshold was set to >0.4. The resulting network was imported into Cytoscape software (Version 3.9.1) (Shannon et al., 2003) for visualization and analysis. The CytoHubba plugin (Chin et al., 2014) was used to assess multiple topological parameters of network nodes, and the top 30 genes for each parameter were selected. The intersecting genes among these rankings were identified as potential hub genes.

### 2.5 Screening and validation of signature genes

Candidate feature genes were screened using two machine learning algorithms: LASSO-logistic regression and support vector machine–recursive feature elimination (SVM-RFE). These models were implemented using the *glmnet* package (Version 4.1–8) (Yang et al., 2022) and the *e1071* package (Version 1.7–13) (Shi et al., 2023) in R, respectively. Receiver operating characteristic (ROC) curves were generated using the pROC package (Version 1.18.5) (Zhang et al., 2022b) and genes with an area under the curve (AUC) greater than 0.7 were considered significant features. All analyses were conducted in both the training and validation datasets.

### 2.6 Machine learning algorithms for constructing gene diagnostic models

To develop a highly accurate and robust diagnostic model for pediatric septic shock, we integrated 12 machine learning algorithms and generated 113 algorithmic combinations to identify models with optimal accuracy and stability. The algorithms included Random Forest (RF), Least Absolute Shrinkage and Selection Operator (Lasso), Ridge, Elastic Net (Enet), Stepwise Generalized Linear Model (StepGLM), Support Vector Machine (SVM), glmBoost, Linear Discriminant Analysis (LDA), Gradient Boosting Machine (GBM), Extreme Gradient Boosting (XGBoost), and Naive Bayes. The modeling process was conducted as follows,all 113 algorithmic combinations were applied to the selected feature genes, and each diagnostic model was trained using 10-fold cross-validation within the training dataset. The performance of all models was subsequently validated using an independent validation set. For each model, the area under the receiver operating characteristic curve (AUC) was calculated across all validation datasets, and the model with the highest mean AUC was identified as the optimal diagnostic model. ROC curves were further plotted across all datasets to evaluate diagnostic accuracy.

### 2.7 Correlation between immune cell infiltration and feature genes

Immune cell infiltration was evaluated using the ssGSEA algorithm implemented in the GSVA package (Version 2.3.1) (Hänzelmann et al., 2013), which quantified the relative abundance of 28 immune cell types in each sample and generated infiltration scores for these immune cells.Differences between groups were analyzed using the Wilcoxon rank-sum test. Spearman correlation analysis was subsequently conducted to assess associations among immune cell types and between signature gene expression and immune cell abundance. Correlation results and interrelationships were visualized using the ggplot2 package.

### 2.8 Gene Enrichment Analysis

Functional enrichment of signature genes was analyzed using the GeneMANIA database (https://genemania.org/) (Warde-Farley et al., 2010), which identified 20 functionally related genes and revealed their potential biological relationships. Based on the median diagnostic model score (nomoScore), patients were classified into high- and low-risk groups. Gene set enrichment analysis (GSEA) was then performed between the two groups to explore enriched Gene Ontology (GO) terms and Kyoto Encyclopedia of Genes and Genomes (KEGG) pathways. Additionally, gene set variation analysis (GSVA) was conducted using the hallmark gene set “h.all.v2025.1. Hs.symbols.gmt” to identify significantly enriched hallmark pathways. Spearman correlations between nomoScore and hallmark enrichment scores were computed, and results were visualized as bar charts using *ggplot2*.

### 2.9 Construction of multifactorial regulatory networks

Because gene expression is tightly controlled by transcriptional and post-transcriptional mechanisms, transcription factors (TFs) regulating the feature genes were predicted using the hTFtarget database (https://guolab.wchscu.cn/hTFtarget/) (Zhang et al., 2020) and the KnockTF database (https://ngdc.cncb.ac.cn/databasecommons/database/id/7140) (Feng et al., 2024) the intersection of TFs predicted by both databases was retained as the final TF set. Similarly, microRNAs (miRNAs) targeting the feature genes were predicted using the miRWalk (http://mirwalk.umm.uni-heidelberg.de/) (Dweep et al., 2014) and miRDB (https://mirdb.org/) (Chen and Wang, 2020) databases, and the overlapping results were used for subsequent analysis. Cytoscape software was employed to visualize the TF–gene and miRNA–gene regulatory networks.

### 2.10 Molecular docking

Information on the relationships between characteristic genes and traditional Chinese medicine compounds was obtained from the COREMINE Medical database (https://coremine.com/medical/#search). Using a significance threshold of P < 0.05, the effective traditional Chinese medicine Chrysanthemum indicum was identified. Subsequently, based on the Traditional Chinese Medicine Systems Pharmacology database and analysis platform (https://www.tcmsp-e.com/index.php), active compounds were systematically screened according to widely adopted criteria in network pharmacology studies, namely, oral bioavailability (OB) ≥30% and drug-likeness (DL)≥ 0.18. Three-dimensional protein structures of characteristic genes were downloaded from the RCSB Protein Data Bank (https://www.rcsb.org/) (Berman et al., 2000), and corresponding ligand structures were obtained from the PubChem database (https://pubchem.ncbi.nlm.nih.gov/) (Wang et al., 2012). Water molecules and native ligands were removed using PyMOL (version 2.6.0a0) (Seeliger and de Groot, 2010), retaining only protein monomers. Molecular docking was performed with CB-DOCK2 (Liu et al., 2022) with default parameters (exhaustiveness = 8 and num_modes = 10) to calculate binding free energies, and the docking interactions were visualized using PyMOL and Discovery Studio Modeling Environment (Release 2019).

## 3 Results

### 3.1 Identification of differentially expressed genes

**FIGURE 1:**
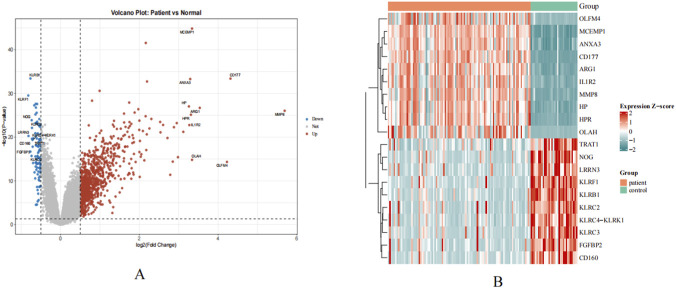
Identify differentially expressed genes **(A)** Volcano plot of differentially expressed genes (DEGs), with red indicating upregulation, blue indicating downregulation, and gray indicating non-differential expression **(B)** Heatmap of the top 10 significantly up- and downregulated DEGs ranked by expression level.

In the GSE26440 dataset, differential expression analysis was conducted between the disease and control groups using the *limma* package. Genes with an p-adjus <0.05 and |log_2_FC| > 0.5 were considered significantly differentially expressed. A total of 930 DEGs were identified, including 835 upregulated and 95 downregulated genes. A volcano plot illustrated the overall gene expression distribution (Figure 1A), while a heatmap displayed the top ten most upregulated and downregulated genes ranked by fold change (Figure 1B).

### 3.2 Analysis of weighted gene Co-expression network

**FIGURE 2:**
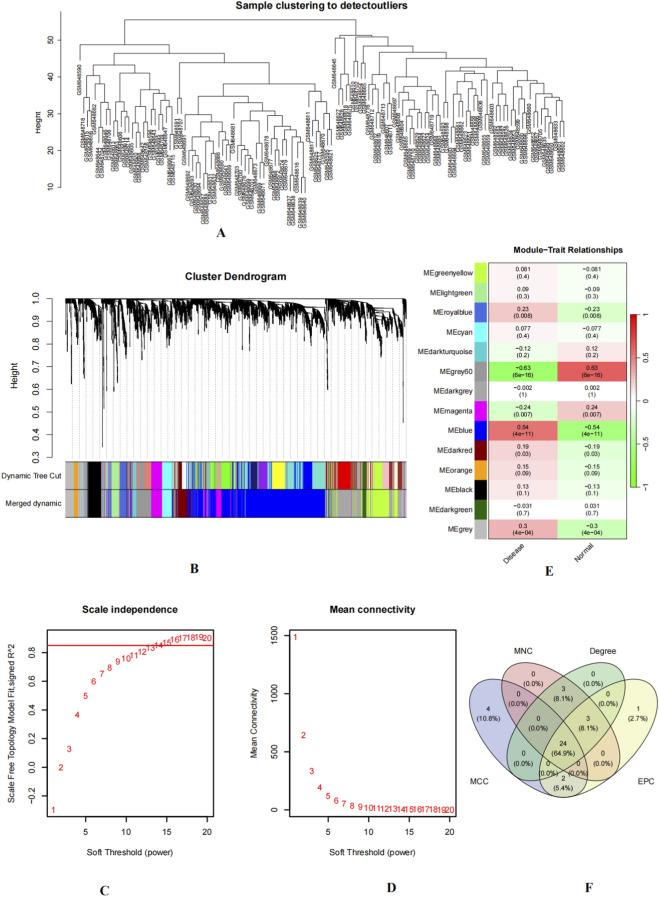
Construct WGCNA and identify hub genes **(A)** Sample clustering dendrogram **(B)** Dendrogram of gene clustering based on topological overlap, with modules represented by different colors **(C,D)** Selection of the optimal soft-thresholding power **(E)** Correlation between modules and traits, where each cell indicates the correlation coefficient and corresponding p-value. The table is color-coded according to the legend **(F)** Venn diagram depicting the overlap of key genes.

The WGCNA package in R was employed to construct a co-expression network using the GSE26440 dataset. Disease status served as the phenotypic trait, and modules were identified through hierarchical clustering and dynamic tree cutting (Figures 2A,B). In this study, the soft-thresholding power (β) was determined using the scale-free topology criterion implemented in the WGCNA package. When β was set to 13, the scale-free topology fit index (*R* ^2^) exceeded the threshold of 0.85, while the mean connectivity of the network remained within a relatively stable and acceptable range. Therefore, this parameter was selected for subsequent network construction to ensure that the co-expression network satisfied both scale-free properties and appropriate gene connectivity (Figures 2C,D). During module identification, the dynamic tree cut method was applied to define gene modules. The minimum module size was set to 50 to avoid instability associated with excessively small modules. Additionally, the deep split parameter was set to two to achieve a sensitive yet biologically meaningful module partitioning. Fourteen distinct modules were detected, each represented by a unique color. Among them, the “MEblue” and “MEgrey60” modules (Figure 2E) showed strong correlations with the pediatric sepsis phenotype (|r| > 0.5, *p* < 0.05). These two modules contained 2,164 genes, which were identified as being highly associated with pediatric septic shock.

### 3.3 Protein–protein interaction network analysis

An intersection analysis between DEGs, WGCNA-derived pathogenic modules, and bacterial lipopolysaccharide–related genes identified 356 overlapping genes. A protein–protein interaction (PPI) network was constructed for these intersecting genes using the STRING database. The resulting network was imported into Cytoscape for visualization and topological analysis. Using the MCC, MNC, Degree, and EPC algorithms, 24 hub genes were identified from the intersection of the four ranking methods (Figure 2F).

### 3.4 Feature gene selection and validation via machine learning algorithms

**FIGURE 3:**
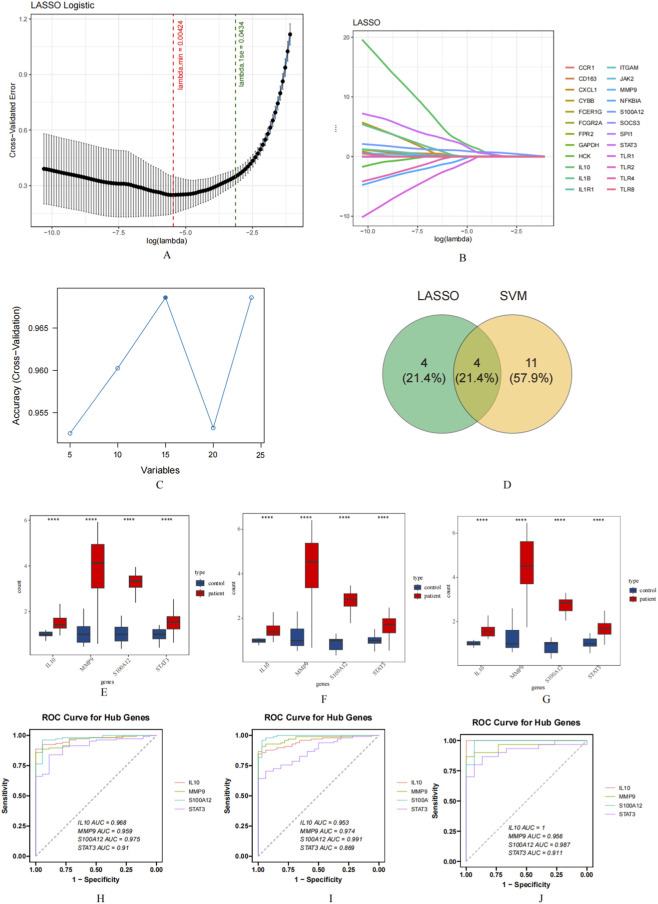
Selection and validation of feature genes **(A)** The LASSO logistic regression coefficient penalty plot is shown. The y-axis (deviance) represents the proportion of residuals explained by the model and illustrates the relationship between the number of feature genes and the proportion of explained residuals (dev). The x-axis corresponds to log (Lambda). The left dashed line marks the position where the cross-validation error reaches its minimum. Based on this value (lambda.min), the corresponding log (Lambda) on the x-axis is determined, and the number of selected feature genes is displayed above the plot, allowing identification of the optimal log (Lambda) **(B)** The LASSO regression path plot is shown. The x-axis represents log (Lambda), and the y-axis represents the gene coefficients. Genes validated at lambda. min were retained according to their coefficient values **(C)** Results of SVM-RFE feature gene selection, with the number of genes on the x-axis and cross-validation accuracy on the y-axis **(D)** Venn diagram of candidate feature genes **(E–G)** Expression boxplots of feature genes in datasets GSE26440, GSE13904, and GSE9692 **(H–J)** The ROC curves demonstrate the significant and consistent expression patterns of the feature genes in the datasets GSE26440, GSE13904, and the validation dataset GSE9692. The x-axis represents the false-positive rate (1-specificity), and the y-axis represents the true-positive rate (sensitivity). The AUC indicates the area under the ROC curve.

Two machine learning methods—LASSO-logistic regression and SVM-RFE—were applied to the GSE26440 dataset to identify 24 key diagnostic genes. Using LASSO logistic regression, we selected the optimal lambda (λ = 0.00424) corresponding to the minimum mean-squared error (MSE) in 10-fold cross-validation. This process generated two plots: the cross-validation error plot (Figure 3A) and the coefficient profile plot (Figure 3B). Eight genes were retained (CXCL1, IL10, IL1B, MMP9, NFKBIA, S100A12, STAT3, and TLR1). Following the same procedure described above, we used the 24 key genes to construct a support vector machine (SVM) model, using the training dataset as input. The SVM algorithm identified 15 genes (S100A12, SOCS3, MMP9, FCER1G, IL10, IL1R1, ITGAM, CD163, TLR2, SPI1, HCK, CCR1, JAK2, CYBB, and STAT3) (Figure 3C). The overlap between the two methods yielded four candidate feature genes: IL10, MMP9, S100A12, and STAT3 (Figure 3D). All four genes were upregulated (Figures 3E–G). ROC analysis demonstrated that all selected genes achieved AUC values above 0.7 in both training and validation datasets (Figures 3H–J), indicating robust diagnostic performance.

### 3.5 Construction and validation of a gene diagnostic model for pediatric septic shock

**FIGURE 4:**
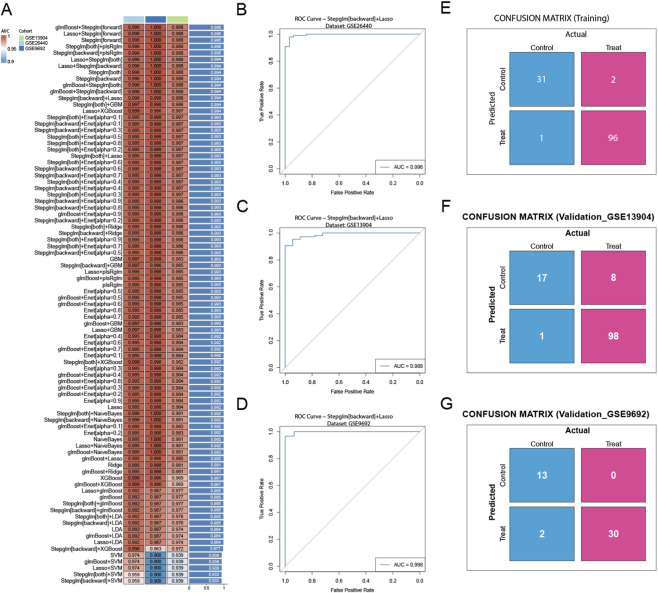
Development and validation of a gene-based diagnostic model for pediatric septic shock. **(A)** Heatmap displaying the AUC values of 113 integrated machine learning models **(B-D)** ROC curves of the optimal diagnostic model (StepGLM [backward] + LASSO) in datasets GSE26440, GSE13904, and GSE9692 **(E-G)** Confusion matrices of the optimal diagnostic model in the training set (GSE26440) and the external validation sets (GSE13904 and GSE9692).

Based on the expression profiles of the four feature genes in the training set and multiple validation datasets, 10-fold cross-validation was performed within the training set to construct 113 final models with optimal performance. During model training, algorithm combinations that retained fewer than two final features were excluded. We subsequently calculated the AUC values of each model across all validation datasets and visualized the results using a heatmap (Figure 4A). Among all models, the Stepglm [backward] + Lasso combination achieved the best overall performance, yielding the highest mean AUC value (0.994), and was therefore identified as the optimal model. Using this model, risk scores were calculated for each patient. ROC analysis showed excellent discrimination in both training (AUC = 0.996, GSE26440) and validation datasets (AUC = 0.988 and 0.998 for GSE13904 and GSE9692, respectively) (Figures 4B–D), confirming the high diagnostic accuracy of the four-gene model. Confusion matrix analyses indicated that the model achieved strong classification performance (Figures 4E–G), with an accuracy of 97.7%, sensitivity of 98.0%, and specificity of 96.9% in the training set. The accuracy remained high in the two independent validation cohorts (95.6% and 92.7%, respectively), supporting the model’s good generalizability across heterogeneous platforms.

### 3.6 Evaluation of immune cell infiltration and correlation analysis

**FIGURE 5:**
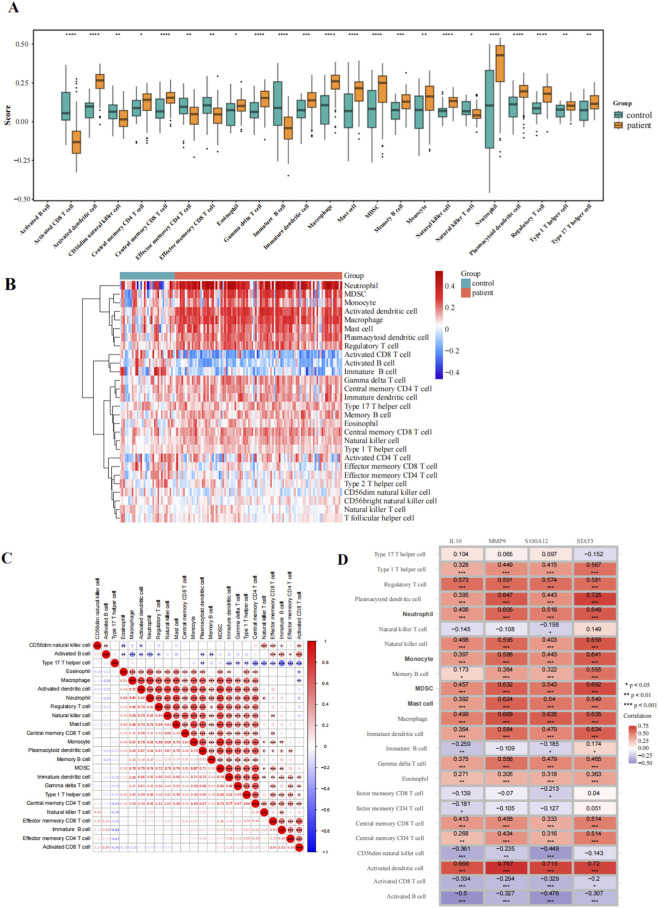
Analysis of the Immune Microenvironment **(A)** Overview of immune cell subset abundance across sample groups (*P < 0.05, **P < 0.01, ***P < 0.001, ****P < 0.0001) **(B)** Heatmap of correlations between different immune cell types. Darker colors indicate stronger correlations; red denotes positive correlations, blue denotes negative correlations **(C)** Comparison of immune cell subset infiltration levels between the control group and pediatric septic shock group (*P < 0.05, **P < 0.01, ***P < 0.001) **(D)** Correlation analysis between gene expression of IL10, MMP9, S100A12, and STAT3 and immune cell subset infiltration abundance (P < 0.05, **P < 0.01, *P < 0.001).

To explore the role of immune infiltration in pediatric septic shock, ssGSEA was applied to the training dataset to quantify the relative abundance of 28 immune cell types. Comparative analysis between disease and control groups revealed significant differences in infiltration levels for multiple immune cells, including activated B cells, activated CD8^+^ T cells, activated dendritic cells, central memory CD8^+^ T cells, γδ T cells, immature B cells, macrophages, neutrophils, plasmacytoid dendritic cells, and regulatory T cells (Figures 5A,B).

Spearman correlation analysis demonstrated a strong positive correlation between MDSCs and monocytes (r = 0.87, *p* < 0.01) and a significant negative correlation between Th17 cells and immature B cells (r = −0.54, *p* < 0.01) (Figure 5C). Furthermore, *MMP9* expression showed a strong positive correlation with activated dendritic cells (r = 0.767, *p* < 0.001) (Figure 5D).

### 3.7 Gene Enrichment Analysis

**FIGURE 6:**
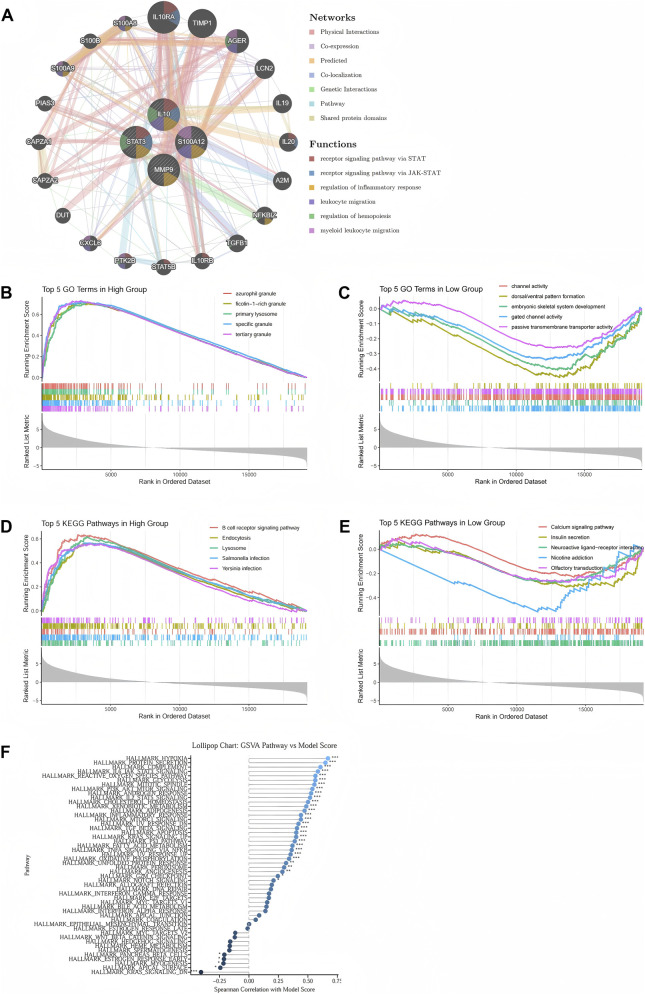
Gene Enrichment Analysis **(A)** Network of feature genes and their top 20 interacting partners. Edge colors represent different interaction types (physical interaction, co-expression, genetic interaction, co-localization, pathway enrichment, or database prediction), while node colors indicate distinct functional annotations. Pediatric sepsis shock samples were stratified into high- and low-score groups based on the median Score of the optimal model **(B,C)** GO enrichment by GSEA in the high- and low-score groups **(D,E)** KEGG enrichment by GSEA in the high- and low-score groups **(F)** Correlation between HALLMARK pathways and Score values.

The GeneMANIA database identified 20 genes functionally associated with the four feature genes, revealing co-expression, co-localization, and shared pathway relationships (Figure 6A). Based on the median diagnostic model score, patients were divided into high- and low-score groups for GSEA enrichment analysis. The top five enriched GO terms in the high-score group (Figure 6B) were “tertiary granule,” “specific granule,” “ficolin-1-rich granule,” “primary lysosome,” and “azurophil granule.” In contrast, the low-score group (Figure 6C) was enriched in “gated channel activity,” “embryonic skeletal system development,” “channel activity,” “passive transmembrane transporter activity,” and “dorsal/ventral pattern formation.“KEGG analysis indicated that the high-score group was mainly enriched in “lysosome,” “*Salmonella* infection,” “B cell receptor signaling pathway,” “endocytosis,” and “*Yersinia* infection” pathways (Figure 6D). The low-score group exhibited enrichment in “neuroactive ligand–receptor interaction,” “nicotine addiction,” “calcium signaling pathway,” “insulin secretion,” and “olfactory transduction” pathways (Figure 6E).

Additionally, GSVA-based analysis of hallmark gene sets revealed 29 positively correlated and five negatively correlated pathways with the Stepglm [backward]+Lasso score (*p* < 0.05) (Figure 6F).

### 3.8 Investigation of multifactorial regulatory networks

**FIGURE 7:**
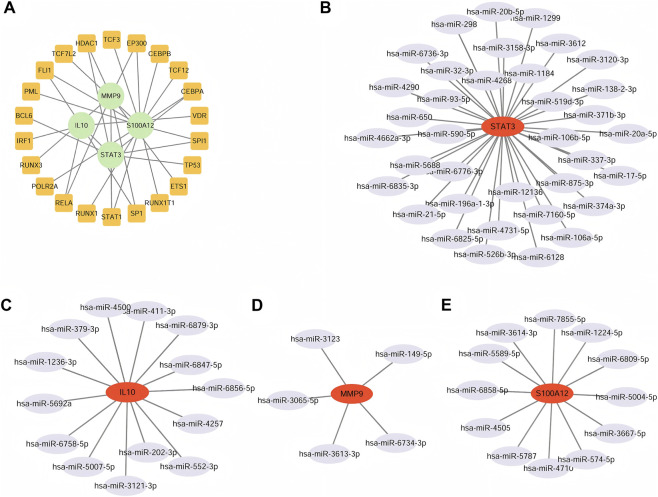
Construction of a multi-factor regulatory network. **(A)** Transcription factor–feature gene interaction network. **(B-E)** miRNA–feature gene interaction network.

By analyzing transcription factor databases, we predicted transcription factors regulating the characteristic genes and further predicted miRNAs regulating these characteristic genes. Using Cytoscape software, we constructed a characteristic gene-transcription factor network comprising 26 nodes and 32 edges (Figure 7A), and a characteristic gene-miRNA network comprising 71 nodes and 67 edges, visualized as shown below (Figures 7B–E).

### 3.9 Screening of traditional Chinese medicine treatment targets for pediatric septic shock

**FIGURE 8:**
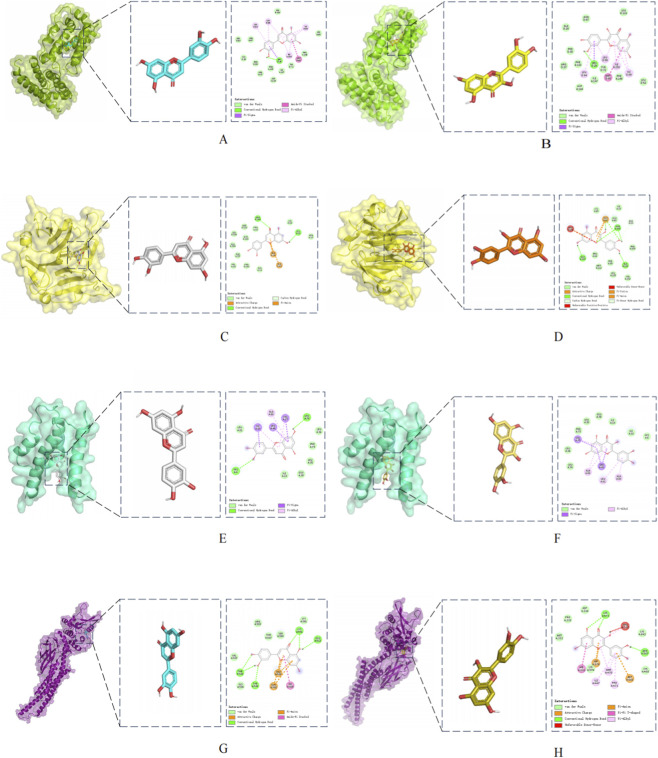
Molecular docking schematics of feature genes and active ingredients from traditional Chinese medicine in pediatric sepsis. **(A-H)** Molecular docking models of pediatric sepsis-associated signature genes (IL10, MMP9, S100A12, STAT3) with bioactive flavonoids luteolin and quercetin. For each complex, the panel (from left to right) depicts the protein-ligand binding conformation, the chemical structure of the ligand, and a two-dimensional diagram of the interaction.

Four characteristic genes were identified as potential therapeutic targets of traditional Chinese medicine. Among these, *Chrysanthemum indicum* was identified as a herb commonly associated with pediatric septic shock treatment. The key bioactive compounds, luteolin and quercetin, were screened using the TCMSP database. Molecular docking of these compounds with the four feature genes (IL10, MMP9, S100A12, and STAT3) demonstrated stable binding affinities (Figures 8A–H).

The binding energies between luteolin and *IL10*, *MMP9*, *S100A12*, and *STAT3* were −7.4, −7.9, −6.4, and −7.4 kcal/mol, respectively, while those of quercetin were −7.5, −8.1, −6.1, and −7.9 kcal/mol, respectively. These results indicate that both luteolin and quercetin exhibit strong binding stability, with *MMP9* showing the most favorable interactions with both compounds.

## 4 Discussion

Pediatric septic shock is a severe, life-threatening condition with clinical manifestations that differ substantially from those in adults, likely reflecting developmental differences in immune and metabolic regulation (Raymond et al., 2017; Weiss et al., 2020). Early diagnosis and prognostic assessment remain challenging. Commonly used biomarkers, such as procalcitonin and C-reactive protein, have limited sensitivity and specificity in pediatric populations (Alqahtani et al., 2016; Hausfater et al., 2021). In parallel, accumulating evidence indicates that molecular signatures and immune cell infiltration patterns are closely linked to sepsis progression and clinical outcomes (Delano and Ward, 2016; Leonard et al., 2024). Therefore, integrating biomarker discovery with immune infiltration profiling holds considerable promise for elucidating the pathophysiology of pediatric septic shock and advancing precision medicine.

Bacterial lipopolysaccharide has long been a central focus of pediatric septic shock research (Carlini et al., 2023); However, comprehensive analyses that jointly apply bioinformatics and machine learning to identify LPS-associated genes in pediatric septic shock remain limited. In this study, integrated analyses identified four LPS-related genes—IL10, MMP9, S100A12, and STAT3—that were associated with pathways relevant to pediatric septic shock. The IL10 gene, located on the long arm of chromosome 1, encodes the anti-inflammatory cytokine interleukin-10 (IL-10). IL-10 is a pleiotropic cytokine that plays a pivotal role in modulating inflammation and maintaining immune homeostasis. It primarily functions as an anti-inflammatory mediator, mitigating excessive immune activation through the JAK1/TYK2–STAT3 signaling cascade (Lee and Kim, 2022). Previous studies have also demonstrated a time-dependent and bidirectional role of IL-10 during sepsis shock. Transient elevation can suppress excessive inflammation, whereas sustained or aberrant upregulation is associated with immunosuppressive phenotypes—such as impaired T-cell function, increased susceptibility to secondary infections, and poor clinical outcomes (Cajander et al., 2024). From the perspective of humoral innate immunity, prolonged or dysregulated IL-10 signaling may accelerate the transition from systemic inflammation to immune exhaustion (Mantovani and Garlanda, 2023). MMP-9,secreted by neutrophils, macrophages, and other immune cells, degrades components of the basement membrane and extracellular matrix (ECM), thereby compromising the vascular endothelial barrier and promoting capillary leakage and tissue edema. Elevated MMP-9 levels have been associated with stronger proinflammatory responses and greater disease severity in sepsis (Vento et al., 2013). Emerging evidence suggests that MMP-9 acts as a key regulatory factor in CD4^+^ T-cell exhaustion within immunological networks. Selective inhibition of MMP-9 using MMP9-in-1 has been shown to significantly restore T-cell function (Wang et al., 2025), supporting the rationale for further investigation of MMP-9 as a potential therapeutic target in pediatric septic shock.

S100A12, a member of the S100 calcium-binding protein family, is primarily expressed in bone marrow cells and damaged cells. It is highly expressed during cellular injury or inflammatory responses, participating in pro-inflammatory regulation by inducing excessive cytokine release, which can trigger septic shock and organ failure in pediatric patients (Uhel et al., 2017). Studies indicate that the S100A12 gene is highly expressed in the peripheral blood of children with sepsis (Lei et al., 2021). As a damage-associated molecular pattern, S100A12 can activate NF-κB and other downstream pathways after receptor engagement, inducing proinflammatory cytokines and chemokines and thereby aggravating endothelial injury and microcirculatory dysfunction (Xia et al., 2024). S100A12 is also closely associated with neutrophil effector mechanisms, including NET formation, oxidative burst, and protease release, which can increase vascular permeability and impair organ perfusion, as supported by proteomic and molecular subtyping studies of sepsis (Zhang and Zhang, 2025). Furthermore, sustained elevation of S100A12 has been linked to dysregulated immune responses, where excessive innate immune activation may drive subsequent T-cell dysfunction and immune exhaustion, ultimately weakening host defense and contributing to adverse outcomes. Overall, the upregulation of S100A12 observed in our study aligns with its established roles in amplifying inflammation, disrupting vascular integrity, and promoting immune imbalance, highlighting its potential as both a prognostic marker and a candidate therapeutic target in pediatric septic shock. In pediatric septic shock, elevated S100A12 levels correlate closely with intensified inflammatory responses and organ damage severity, positioning it as a potential biomarker for inflammation severity and prognosis. STAT3 is a key member of the signal transduction and transcription activator family, participating in both proinflammatory responses and playing a central role in anti-inflammatory signaling pathways such as IL-10 (Hutchins et al., 2012). Previous studies confirm that inhibiting STAT3 activity may alter organ inflammatory responses in LPS-induced sepsis models. In pediatric septic shock, LPS activates the JAK/STAT pathway, where STAT3 hyperactivation leads to excessive inflammatory factor release and coexisting immunosuppression, thereby worsening disease progression (Schrijver et al., 2019; Clere-Jehl et al., 2020). Based on the aforementioned four characteristic genes, we developed a diagnostic model in the GSE26440 dataset. Validation in a separate dataset yielded AUC values exceeding 0.9, confirming the model’s robust diagnostic efficacy. Developing and validating a gene-based diagnostic model for pediatric septic shock holds significantimplications for early diagnosis and timely intervention, thereby improving patient outcomes.

Patients were stratified into high- and low-score groups according to the median value of the optimal gene-based diagnostic model score, followed by KEGG, GO, and HALLMARK pathway analyses. The results revealed that the high-score group exhibited a phenotype characterized by inflammatory and immune hyperactivation, whereas the low-score group displayed features consistent with homeostatic maintenance and tissue repair. These divergent pathway profiles suggest substantial immunometabolic heterogeneity across pediatric septic shock, which may influence prognosis and therapeutic responsiveness.

In pediatric septic shock, innate immune cells—including dendritic cells (DCs), macrophages, neutrophils, and monocytes—are broadly activated, reflecting a vigorous immune response to infection. Although these cells play pivotal roles in pathogen recognition, antigen presentation, and cytokine secretion, their excessive activation can precipitate tissue injury and multiorgan dysfunction (Gao et al., 2025). Notably, myeloid-derived suppressor cells (MDSCs) were significantly elevated in the shock group, suggesting activation of immunosuppressive mechanisms. MDSCs exacerbate immunosuppression by suppressing T-cell effector activity and promoting regulatory T-cell (Treg) expansion (Huang et al., 2024). Consistent with previous findings (Drewry et al., 2014), our analysis demonstrated a marked reduction in peripheral T and B lymphocytes in pediatric septic shock, with persistent lymphopenia strongly associated with secondary infections, organ failure, and mortality. Moreover, the expansion of Th1 and Th17 cells reflects a sustained proinflammatory state, potentially amplifying tissue damage (Liu et al., 2024). Collectively, these results underscore that pediatric septic shock is characterized by concurrent overactivation of innate immunity and dysfunction of adaptive immunity, leading to a complex state of immune imbalance. This coexistence of hyperinflammation and immunosuppression may represent a fundamental mechanism underlying persistent infection susceptibility and poor clinical outcomes in these patients.

This study further identified four bacterial lipopolysaccharide-associated characteristic genes—IL10, MMP9, STAT3, and S100A12—that participate in inflammatory signaling and immune regulation through intricate regulatory networks. These findings provide novel molecular evidence for early diagnosis and targeted treatment of pediatric sepsis shock. STAT3, a central mediator of signal transduction, is modulated by multiple miRNAs including miR-20b-5p, miR-93-5p, and miR-106a-5p, and plays a crucial role in immune dysregulation and inflammatory amplification (Tang and Luo, 2018; Dragomir et al., 2023). Importantly, inhibition of miR-93-5p has been shown to improve survival and restore immune function in sepsis models. IL-10, a principal anti-inflammatory cytokine, is subject to dual regulation by transcription factors such as RELA and SPI1, as well as miRNAs including miR-202-3p, miR-3121-3p, and miR-5692a, maintaining the balance between inflammation and immune suppression (Zhang et al., 2022). MMP9, strongly linked to tissue destruction and immune cell migration, exhibited significant correlations with RUNX1, TP53, and SP1, and is regulated by miRNAs such as miR-149-5p, which directly targets MMP9 and modulates cell migration and extracellular matrix remodeling (Peng et al., 2020). S100A12, a neutrophil activation–associated protein, is finely tuned by miRNAs including miR-4710, miR-574-5p, and miR-4505. Of note, miR-574-5p has been reported to attenuate LPS-induced inflammation, whereas miR-30a suppresses S100A12-mediated microglial activation (Dong and Wang, 2019, 574; He et al., 2021).

In recent years, traditional Chinese medicine (TCM) has attracted increasing attention as a potential adjunctive approach for sepsis shock management (Ji et al., 2025). Chrysanthemum indicum, a well-known TCM herb, possesses heat-clearing, detoxifying, and anti-inflammatory properties. Its major bioactive compounds, luteolin and quercetin, have been shown to mitigate septic shock progression by modulating diverse signaling pathways and physiological processes (Aziz et al., 2018; Karimi et al., 2021). Quercetin markedly suppresses the production of proinflammatory mediators such as TNF-α, IL-6, and HMGB1 by inhibiting the TLR/NF-κB and MAPK signaling pathways (Cui et al., 2019). Simultaneously, it enhances antioxidant enzyme activities, including SOD and CAT, thereby reducing oxidative stress, apoptosis, and tissue injury (Sun et al., 2021). Luteolin, by inhibiting HMGB1-mediated inflammation and regulating the MAPK/NF-κB cascade, not only reduces systemic inflammation but also exerts neuroprotective effects against septic encephalopathy, improving cognitive outcomes (Dickson and Lehmann, 2019). Through molecular docking analyses, our study further confirmed the stable binding affinity between luteolin, quercetin, and the identified characteristic genes, providing robust evidence supporting personalized therapeutic strategies for pediatric septic shock.

## 5 Conclusion

This study integrated bioinformatics and machine learning approaches to identify four key LPS-associated genes—IL10, MMP9, S100A12, and STAT3—and explored their diagnostic potential, biological regulatory mechanisms, and immunological features in pediatric septic shock. These findings enhance our understanding of immune dysregulation during disease progression and offer valuable insights into the development of diagnostic biomarkers and precision therapeutic targets for pediatric sepsis.

Nevertheless, several limitations must be acknowledged. First, this study was based on publicly available datasets and employed integrated bioinformatics and machine-learning analyses; however, experimental validation and confirmation in clinical specimens have not yet been performed. Therefore, while we identified LPS-associated differentially expressed genes and described related immune infiltration patterns, these observations should be regarded primarily as computational evidence, and their direct implications for clinical application remain limited. In future work, we plan to validate these findings in animal models and in well-characterized patient samples, which will strengthen the evidence base and improve the translational relevance of our results. Second, differences in sample sources, experimental conditions, and data processing procedures across studies may have a certain impact on model performance. Future studies are still warranted to further validate the robustness of the model in larger, multicenter cohorts, thereby enhancing its potential for clinical application. Meanwhile, different confidence interval thresholds may influence the network structure; therefore, further validation of the stability of key genes under more stringent threshold settings is also needed to improve the robustness and reproducibility of the results. Additionally, multidimensional patient-level metadata-such as demographic information, pathogen type, and therapeutic interventions-carry important mechanistic significance. Integrating these variables into differential expression analyses would provide a more comprehensive understanding of disease processes. However, due to the design and data limitations of the current study, such metadata could not be incorporated. Future research should aim to obtain and utilize these clinical variables to advance mechanistic insights and facilitate translation of findings into diagnostic and therapeutic applications.

## Data Availability

The datasets presented in this study can be found in online repositories. The names of the repository/repositories and accession number(s) can be found in the article/Supplementary Material.

## References

[B1] Alqahtani M. F. Smith C. M. Weiss S. L. Dawson S. Ralay Ranaivo H. Wainwright M. S. (2016). Evaluation of new diagnostic biomarkers in pediatric sepsis: matrix metalloproteinase-9, tissue inhibitor of metalloproteinase-1, mid-regional pro-atrial natriuretic peptide, and adipocyte fatty-acid binding protein. PLOS One 11, e0153645. 10.1371/journal.pone.0153645 27089280 PMC4835068

[B2] Aziz N. Kim M.-Y. Cho J. Y. (2018). Anti-inflammatory effects of luteolin: a review of *in vitro,*, *in vivo,*, and *in silico* studies. J. Ethnopharmacol. 225, 342–358. 10.1016/j.jep.2018.05.019 29801717

[B3] Barrett T. Suzek T. O. Troup D. B. Wilhite S. E. Ngau W.-C. Ledoux P. (2005). NCBI GEO: mining millions of expression Profiles—Database and tools. Nucleic Acids Res. 33, D562–D566. 10.1093/nar/gki022 15608262 PMC539976

[B4] Berman H. M. Westbrook J. Feng Z. Gilliland G. Bhat T. N. Weissig H. (2000). The protein data bank. Nucleic Acids Res. 28, 235–242. 10.1093/nar/28.1.235 10592235 PMC102472

[B5] Biswas S. K. Lopez-Collazo E. (2009). Endotoxin tolerance: new mechanisms, molecules and clinical significance. Trends Immunol. 30, 475–487. 10.1016/j.it.2009.07.009 19781994

[B6] Cajander S. Kox M. Scicluna B. P. Weigand M. A. Mora R. A. Flohé S. B. (2024). Profiling the dysregulated immune response in sepsis: overcoming challenges to achieve the goal of precision medicine. Lancet, Respir. Med. 12, 305–322. 10.1016/S2213-2600(23)00330-2 38142698

[B7] Carlini V. Noonan D. M. Abdalalem E. Goletti D. Sansone C. Calabrone L. (2023). The multifaceted nature of IL-10: regulation, role in immunological homeostasis and its relevance to cancer, COVID-19 and post-COVID conditions. Front. Immunol. 14, 1161067. 10.3389/fimmu.2023.1161067 37359549 PMC10287165

[B8] Chen H. Boutros P. C. (2011). VennDiagram: a package for the generation of highly-customizable venn and euler diagrams in R. BMC Bioinf 12, 35. 10.1186/1471-2105-12-35 21269502 PMC3041657

[B9] Chen Y. Wang X. (2020). miRDB: an online database for prediction of functional microRNA targets. Nucleic Acids Res. 48, D127–D131. 10.1093/nar/gkz757 31504780 PMC6943051

[B10] Chin C.-H. Chen S.-H. Wu H.-H. Ho C.-W. Ko M.-T. Lin C.-Y. (2014). cytoHubba: identifying hub objects and sub-networks from complex interactome. BMC Syst. Biol. 8 (Suppl. 4), S11. 10.1186/1752-0509-8-S4-S11 25521941 PMC4290687

[B11] Clere-Jehl R. Mariotte A. Meziani F. Bahram S. Georgel P. Helms J. (2020). JAK-STAT targeting offers novel therapeutic opportunities in sepsis. Trends Mol. Med. 26, 987–1002. 10.1016/j.molmed.2020.06.007 32631717

[B12] Cui W. Hu G. Peng J. Mu L. Liu J. Qiao L. (2019). Quercetin exerted protective effects in a rat model of sepsis *via* inhibition of reactive oxygen species (ROS) and downregulation of high mobility group box 1 (HMGB1) protein expression. Med. Sci. Monit. Int. Med. J. Exp. Clin. Res. 25, 5795–5800. 10.12659/MSM.916044 31377749 PMC6691752

[B13] Davis A. P. Wiegers T. C. Johnson R. J. Sciaky D. Wiegers J. Mattingly C. J. (2023). Comparative toxicogenomics database (CTD): update 2023. Nucleic Acids Res. 51, D1257–D1262. 10.1093/nar/gkac833 36169237 PMC9825590

[B14] Delano M. J. Ward P. A. (2016). The immune system’s role in sepsis progression, resolution, and long-term outcome. Immunol. Rev. 274, 330–353. 10.1111/imr.12499 27782333 PMC5111634

[B15] Dickson K. Lehmann C. (2019). Inflammatory response to different toxins in experimental sepsis models. Int. J. Mol. Sci. 20, 4341. 10.3390/ijms20184341 31491842 PMC6770119

[B16] Dm F. Ja K. (2023). Endotoxic septic shock: diagnosis and treatment. Int. Journal Molecular Sciences 24, 16185. 10.3390/ijms242216185 38003374 PMC10671446

[B17] Dong N. Wang Y. (2019). MiR-30a regulates S100A12-induced retinal microglial activation and inflammation by targeting NLRP3. Curr. Eye Res. 44, 1236–1243. 10.1080/02713683.2019.1632350 31199706

[B18] Dragomir M. P. Fuentes-Mattei E. Winkle M. Okubo K. Bayraktar R. Knutsen E. (2023). Anti–miR-93-5p therapy prolongs sepsis survival by restoring the peripheral immune response. J. Clin. Invest. 133, e158348. 10.1172/JCI158348 37261908 PMC10348769

[B19] Drewry A. M. Samra N. Skrupky L. P. Fuller B. M. Compton S. M. Hotchkiss R. S. (2014). Persistent lymphopenia after diagnosis of sepsis predicts mortality. Shock (augusta Ga) 42, 383–391. 10.1097/SHK.0000000000000234 25051284 PMC4362626

[B20] Dweep H. Gretz N. Sticht C. (2014). miRWalk database for miRNA-target interactions. Methods Mol. Biol. Clift. N.J. 1182, 289–305. 10.1007/978-1-4939-1062-5_25 25055920

[B21] Feng C. Song C. Song S. Zhang G. Yin M. Zhang Y. (2024). KnockTF 2.0: a comprehensive gene expression profile database with knockdown/knockout of transcription (co-)factors in multiple species. Nucleic Acids Res. 52, D183–D193. 10.1093/nar/gkad1016 37956336 PMC10767813

[B22] Gao X. Cai S. Li X. Wu G. (2025). Sepsis-induced immunosuppression: mechanisms, biomarkers and immunotherapy. Front. Immunol. 16, 1577105. 10.3389/fimmu.2025.1577105 40364841 PMC12069044

[B23] Gu Z. (2022). Complex heatmap visualization. Imeta 1, e43. 10.1002/imt2.43 38868715 PMC10989952

[B24] Gustavsson E. K. Zhang D. Reynolds R. H. Garcia-Ruiz S. Ryten M. (2022). Ggtranscript: an R package for the visualization and interpretation of transcript isoforms using ggplot2. Bioinform. Oxf. Engl. 38, 3844–3846. 10.1093/bioinformatics/btac409 35751589 PMC9344834

[B25] Hänzelmann S. Castelo R. Guinney J. (2013). GSVA: gene set variation analysis for microarray and RNA-Seq data. BMC Bioinf. 14, 7. 10.1186/1471-2105-14-7 23323831 PMC3618321

[B26] Hausfater P. Robert Boter N. Morales Indiano C. Cancella de Abreu M. Marin A. M. Pernet J. (2021). Monocyte distribution width (MDW) performance as an early sepsis indicator in the emergency department: comparison with CRP and procalcitonin in a multicenter international european prospective study. Crit. Care (lond. Engl.) 25, 227. 10.1186/s13054-021-03622-5 34193208 PMC8247285

[B27] He B. Zhou W. Rui Y. Liu L. Chen B. Su X. (2021). MicroRNA-574-5p attenuates acute respiratory distress syndrome by targeting HMGB1. Am. J. Respir. Cell Mol. Biol. 64, 196–207. 10.1165/rcmb.2020-0112OC 33202146 PMC7874400

[B28] Hotchkiss R. S. Monneret G. Payen D. (2013). Sepsis-induced immunosuppression: from cellular dysfunctions to immunotherapy. Nat. Rev. Immunol. 13, 862–874. 10.1038/nri3552 24232462 PMC4077177

[B29] Huang S. Liu D. Han L. Deng J. Wang Z. Jiang J. (2024). Decoding the potential role of regulatory T cells in sepsis-induced immunosuppression. Eur. J. Immunol. 54, e2350730. 10.1002/eji.202350730 38430202

[B30] Hutchins A. P. Poulain S. Miranda-Saavedra D. (2012). Genome-wide analysis of STAT3 binding *in vivo* predicts effectors of the anti-inflammatory response in macrophages. Blood 119, e110–e119. 10.1182/blood-2011-09-381483 22323479

[B31] Ji Y. Song H. Li L. (2025). Traditional Chinese medicine for sepsis: advancing from evidence to innovative drug discovery. Crit. Care (lond. Engl.) 29, 193. 10.1186/s13054-025-05441-4 40375087 PMC12080179

[B32] Jiang H. Ren Y. Yu J. Hu S. Zhang J. (2023). Analysis of lactate metabolism-related genes and their association with immune infiltration in septic shock *via* bioinformatics method. Front. Genet. 14, 1223243. 10.3389/fgene.2023.1223243 37564869 PMC10410269

[B33] Karimi A. Naeini F. Asghari Azar V. Hasanzadeh M. Ostadrahimi A. Niazkar H. R. (2021). A comprehensive systematic review of the therapeutic effects and mechanisms of action of quercetin in sepsis. Phytomed. Int. J. Phytother. Phytopharm. 86, 153567. 10.1016/j.phymed.2021.153567 33940332

[B34] Langfelder P. Horvath S. (2008). WGCNA: an R package for weighted correlation network analysis. BMC Bioinf 9, 559. 10.1186/1471-2105-9-559 19114008 PMC2631488

[B35] Lee H. S. Kim W. J. (2022). The role of matrix metalloproteinase in inflammation with a focus on infectious diseases. Int. J. Mol. Sci. 23, 10546. 10.3390/ijms231810546 36142454 PMC9500641

[B36] Lei W. Liu D. Sun M. Lu C. Yang W. Wang C. (2021). Targeting STAT3: a crucial modulator of sepsis. J. Cell. Physiol. 236, 7814–7831. 10.1002/jcp.30394 33885157

[B37] Leonard S. Guertin H. Odoardi N. Miller M. R. Patel M. A. Daley M. (2024). Pediatric sepsis inflammatory blood biomarkers that correlate with clinical variables and severity of illness scores. J. Inflamm. Lond. Engl. 21, 7. 10.1186/s12950-024-00379-w 38454423 PMC10921642

[B38] Liu S. Wang Z. Zhu R. Wang F. Cheng Y. Liu Y. (2021). Three differential expression analysis methods for RNA sequencing: limma, EdgeR, DESeq2. J. Vis. Exp. Jove (175), e62528. 10.3791/62528 34605806

[B39] Liu Y. Yang X. Gan J. Chen S. Xiao Z.-X. Cao Y. (2022). CB-Dock2: improved protein-ligand blind docking by integrating cavity detection, docking and homologous template fitting. Nucleic Acids Res. 50, W159–W164. 10.1093/nar/gkac394 35609983 PMC9252749

[B40] Liu X. Chen L. Peng W. Deng H. Ni H. Tong H. (2024). Th17/treg balance: the bloom and wane in the pathophysiology of sepsis. Front. Immunol. 15, 1356869. 10.3389/fimmu.2024.1356869 38558800 PMC10978743

[B41] Lu Y.-C. Yeh W.-C. Ohashi P. S. (2008). LPS/TLR4 signal transduction pathway. Cytokine 42, 145–151. 10.1016/j.cyto.2008.01.006 18304834

[B42] Mantovani A. Garlanda C. (2023). Humoral innate immunity and acute-phase proteins. N. Engl. J. Med. 388, 439–452. 10.1056/NEJMra2206346 36724330 PMC9912245

[B43] Mazgaeen L. Gurung P. (2020). Recent advances in lipopolysaccharide recognition systems. Int. J. Mol. Sci. 21, 379. 10.3390/ijms21020379 31936182 PMC7013859

[B44] Park B. S. Lee J.-O. (2013). Recognition of lipopolysaccharide pattern by TLR4 complexes. Exp. Mol. Med. 45, e66. 10.1038/emm.2013.97 24310172 PMC3880462

[B45] Peng W. Li T. Pi S. Huang L. Liu Y. (2020). Suppression of circular RNA circDHCR24 alleviates aortic smooth muscle cell proliferation and migration by targeting miR-149-5p/MMP9 axis. Biochem. Biophys. Res. Commun. 529, 753–759. 10.1016/j.bbrc.2020.06.067 32736703

[B46] Raymond S. L. López M. C. Baker H. V. Larson S. D. Efron P. A. Sweeney T. E. (2017). Unique transcriptomic response to sepsis is observed among patients of different age groups. PLOS One 12, e0184159. 10.1371/journal.pone.0184159 28886074 PMC5590890

[B47] Rudd K. E. Johnson S. C. Agesa K. M. Shackelford K. A. Tsoi D. Kievlan D. R. (2020). Global, regional, and national sepsis incidence and mortality, 1990-2017: analysis for the global burden of disease study. Lancet (lond. Engl.) 395, 200–211. 10.1016/S0140-6736(19)32989-7 31954465 PMC6970225

[B48] Sanchez-Pinto L. N. Bennett T. D. DeWitt P. E. Russell S. Rebull M. N. Martin B. (2024). Development and validation of the Phoenix criteria for pediatric sepsis and septic shock. JAMA 331, 675. 10.1001/jama.2024.0196 38245897 PMC10900964

[B49] Schlapbach L. J. Watson R. S. Sorce L. R. Argent A. C. Menon K. Hall M. W. (2024). International consensus criteria for pediatric sepsis and septic shock. JAMA 331, 665–674. 10.1001/jama.2024.0179 38245889 PMC10900966

[B50] Schrijver I. T. Théroude C. Roger T. (2019). Myeloid-derived suppressor cells in sepsis. Front. Immunol. 10, 327. 10.3389/fimmu.2019.00327 30873175 PMC6400980

[B51] Seeliger D. de Groot B. L. (2010). Ligand docking and binding site analysis with PyMOL and autodock/vina. J. Comput.-Aided Mol. Des. 24, 417–422. 10.1007/s10822-010-9352-6 20401516 PMC2881210

[B52] Shannon P. Markiel A. Ozier O. Baliga N. S. Wang J. T. Ramage D. (2003). Cytoscape: a software environment for integrated models of biomolecular interaction networks. Genome Res. 13, 2498–2504. 10.1101/gr.1239303 14597658 PMC403769

[B53] Shi H. Yuan X. Liu G. Fan W. (2023). Identifying and validating GSTM5 as an immunogenic gene in diabetic foot ulcer using bioinformatics and machine learning. J. Inflamm. Res. 16, 6241–6256. 10.2147/JIR.S442388 38145013 PMC10748866

[B54] Singer M. Deutschman C. S. Seymour C. W. Shankar-Hari M. Annane D. Bauer M. (2016). The third international consensus definitions for sepsis and septic shock (sepsis-3). JAMA 315, 801–810. 10.1001/jama.2016.0287 26903338 PMC4968574

[B55] Sun J. Zhang H. Liu D. Cui L. Wang Q. Gan L. (2021). A functional variant of CXCL16 is associated with predisposition to sepsis and MODS in trauma patients: genetic association studies. Front. Genet. 12, 720313. 10.3389/fgene.2021.720313 34539750 PMC8446271

[B56] Szklarczyk D. Kirsch R. Koutrouli M. Nastou K. Mehryary F. Hachilif R. (2022). The STRING database in 2023: Protein–Protein association networks and functional enrichment analyses for any sequenced genome of interest. Nucleic Acids Res. 51, D638–D646. 10.1093/nar/gkac1000 36370105 PMC9825434

[B57] Tang J. Luo L. (2018). MicroRNA-20b-5p inhibits platelet-derived growth factor-induced proliferation of human fetal airway smooth muscle cells by targeting signal transducer and activator of transcription 3. Biomed. Pharmacother. 102, 34–40. 10.1016/j.biopha.2018.03.015 29549727

[B58] Uhel F. Azzaoui I. Grégoire M. Pangault C. Dulong J. Tadié J.-M. (2017). Early expansion of circulating granulocytic myeloid-derived suppressor cells predicts development of nosocomial infections in patients with sepsis. Am. J. Respir. Crit. Care Med. 196, 315–327. 10.1164/rccm.201606-1143OC 28146645

[B59] Van Der Poll T. Shankar-Hari M. Wiersinga W. J. (2021). The immunology of sepsis. Immunity 54, 2450–2464. 10.1016/j.immuni.2021.10.012 34758337

[B60] Vento G. Lio A. Tirone C. Aurilia C. Tana M. Piras A. (2013). Association of high levels of α-defensins and S100A proteins with candida mannan detection in bronchoalveolar lavage fluid of preterm neonates. Pediatr. Res. 74, 19–25. 10.1038/pr.2013.60 23575874

[B61] Wang X. Quinn P. J. (2010). Endotoxins: lipopolysaccharides of gram-negative bacteria. Sub-cell. Biochem. 53, 3–25. 10.1007/978-90-481-9078-2_1 20593260

[B62] Wang Y. Xiao J. Suzek T. O. Zhang J. Wang J. Zhou Z. (2012). PubChem’s BioAssay database. Nucleic Acids Res. 40, D400–D412. 10.1093/nar/gkr1132 22140110 PMC3245056

[B63] Wang M. Feng J. Zhou D. Wang J. (2023). Bacterial lipopolysaccharide-induced endothelial activation and dysfunction: a new predictive and therapeutic paradigm for sepsis. Eur. J. Med. Res. 28, 339. 10.1186/s40001-023-01301-5 37700349 PMC10498524

[B64] Wang X. Ning J. Zhou L. Li H. Cui J. Wang J. (2025). Targeting matrix Metalloproteinase-9 to alleviate T cell exhaustion and improve sepsis prognosis. Research Wash D C, 8, 0996. 10.34133/research.0996 41306770 PMC12645450

[B65] Warde-Farley D. Donaldson S. L. Comes O. Zuberi K. Badrawi R. Chao P. (2010). The GeneMANIA prediction server: biological network integration for gene prioritization and predicting gene function. Nucleic Acids Res. 38, W214–W220. 10.1093/nar/gkq537 20576703 PMC2896186

[B66] Weiss S. L. Peters M. J. Alhazzani W. Agus M. S. D. Flori H. R. Inwald D. P. (2020). Surviving sepsis campaign international guidelines for the management of septic shock and sepsis-associated organ dysfunction in children. Pediatr. Crit. Care Med. J. Soc. Crit. Care Med. World Fed. Pediatr. Intensive Crit. Care Soc. 21, e52–e106. 10.1097/PCC.0000000000002198 32032273

[B67] Xia P. Ji X. Yan L. Lian S. Chen Z. Luo Y. (2024). Roles of S100A8, S100A9 and S100A12 in infection, inflammation and immunity. Immunology 171, 365–376. 10.1111/imm.13722 38013255

[B68] Yamamoto M. Akira S. (2010). “Lipid a receptor TLR4-mediated signaling pathways,” in Lipid A in cancer therapy. Editor Jeannin J.-F. (New York, NY: Springer), 59–68. 10.1007/978-1-4419-1603-7_6 20665200

[B69] Yang L. Qu Q. Hao Z. Sha K. Li Z. Li S. (2022). Powerful identification of large quantitative trait loci using genome-wide R/glmnet-based regression. J. Hered. 113, 472–478. 10.1093/jhered/esac006 35134967

[B70] Zhang G. Zhang K. (2025). Screening and identification of neutrophil extracellular trap-related diagnostic biomarkers for pediatric sepsis by machine learning. Inflammation 48, 212–222. 10.1007/s10753-024-02059-6 38795170

[B71] Zhang Q. Liu W. Zhang H.-M. Xie G.-Y. Miao Y.-R. Xia M. (2020). hTFtarget: a comprehensive database for regulations of human transcription factors and their targets. Genomics Proteomics Bioinforma. 18, 120–128. 10.1016/j.gpb.2019.09.006 32858223 PMC7647694

[B72] Zhang Y. Cheng J. Su Y. Li M. Wen J. Li S. (2022). Cordycepin induces M1/M2 macrophage polarization to attenuate the liver and lung damage and immunodeficiency in immature mice with sepsis via NF-κB/p65 inhibition. J. Pharm. Pharmacol. 74, 227–235. 10.1093/jpp/rgab162 34850068

[B73] Zhang Z. Yang Z. Chen M. Li Y. (2022). Compound heterozygous protein C deficiency with pulmonary embolism caused by a novel PROC gene mutation: case report and literature review. Med. Baltim. 101, e31221. 10.1097/MD.0000000000031221 36281079 PMC9592271

