# Supplementary figures and images for: Systematic analysis of bacterial lipopolysaccharide-related genes and immune cell infiltration characteristics in pediatric septic shock using integrated bioinformatics and machine learning approaches
Source: Front Bioinform. 2026 May 29;6:1729982. doi: 10.3389/fbinf.2026.1729982 (PMC13261020; doi:10.3389/fbinf.2026.1729982)

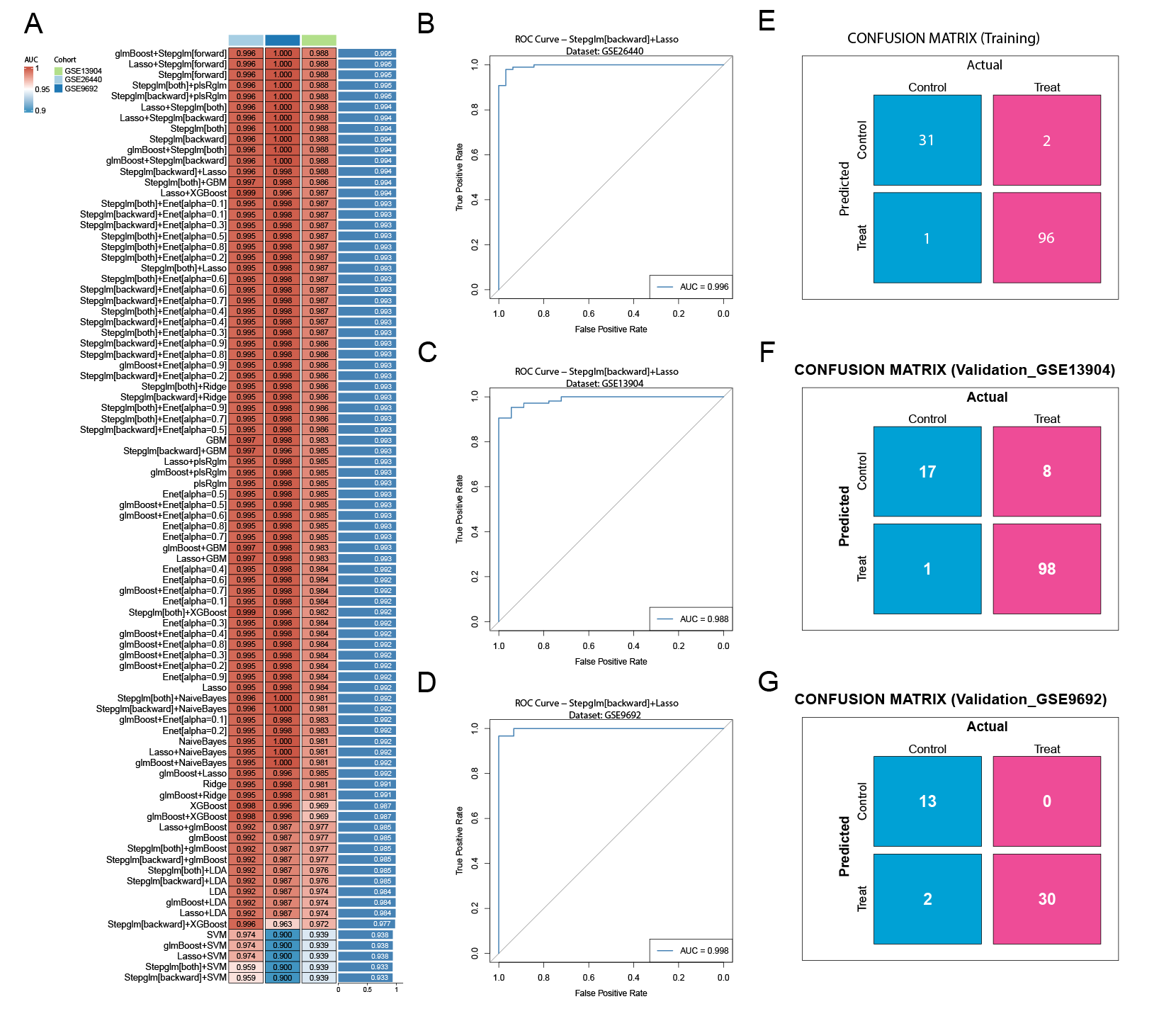

Supplement: Supplementary file 1 [file Image5.png]

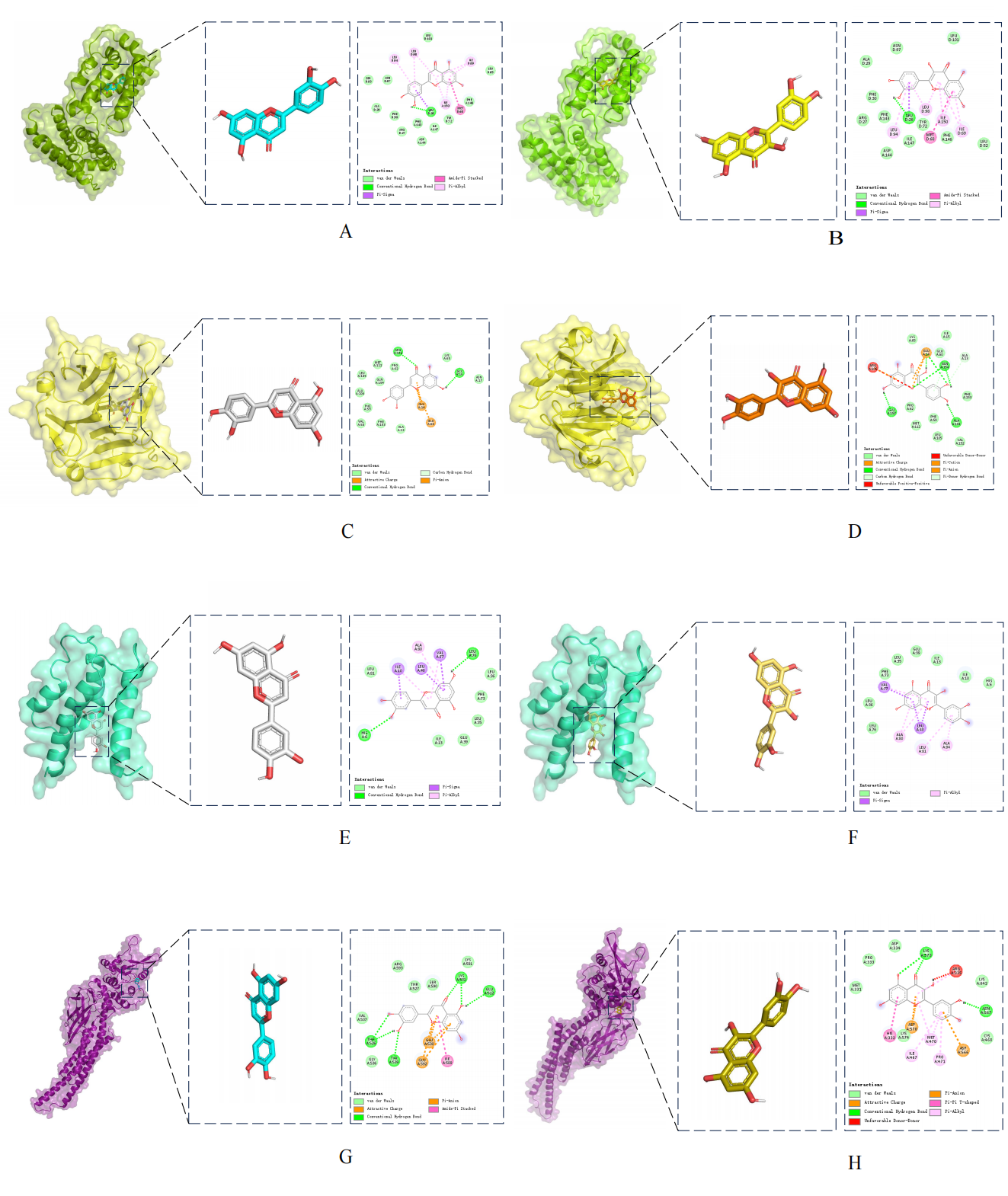

Supplement: Supplementary file 2 [file Image4.png]

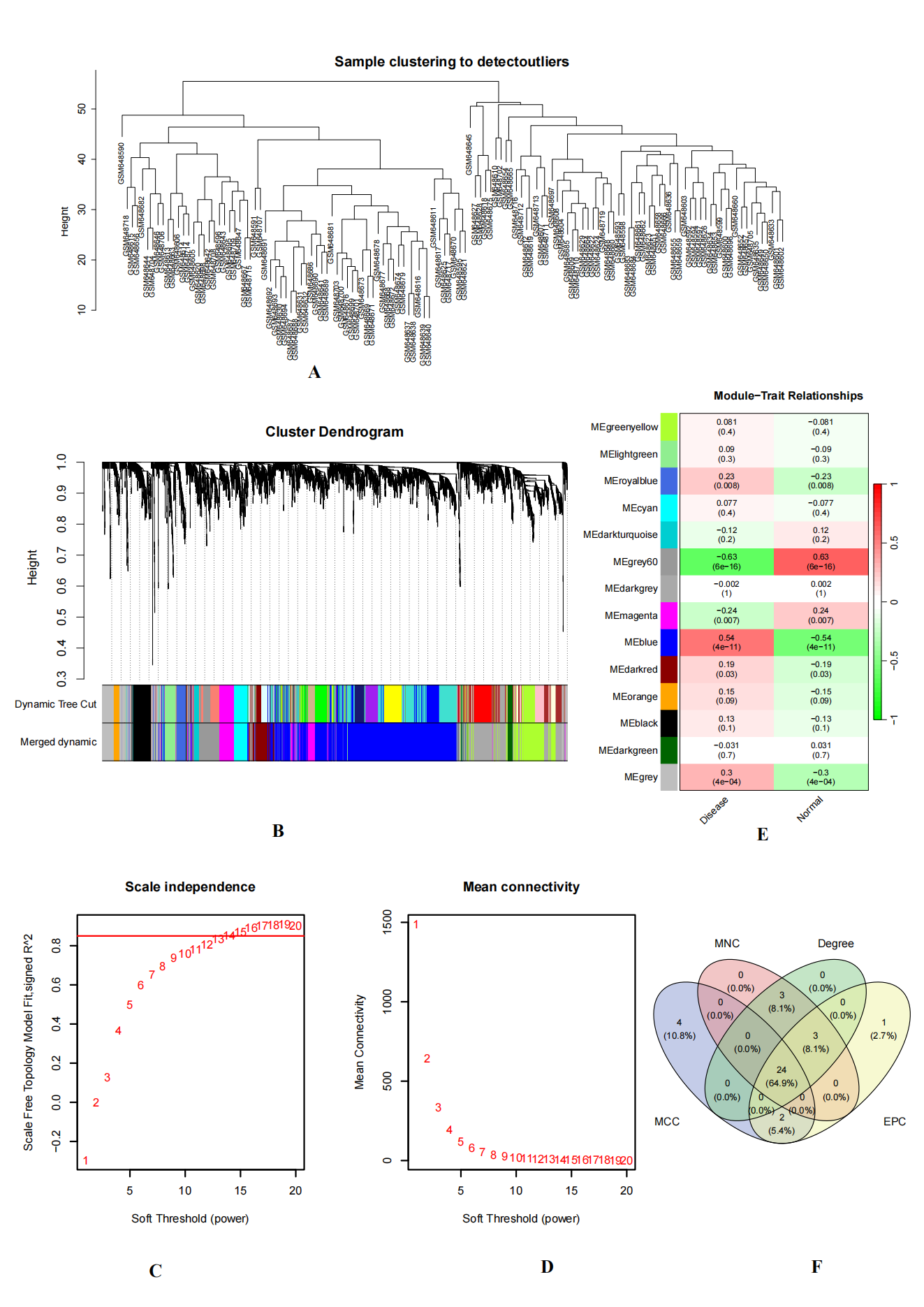

Supplement: Supplementary file 3 [file Image7.png]

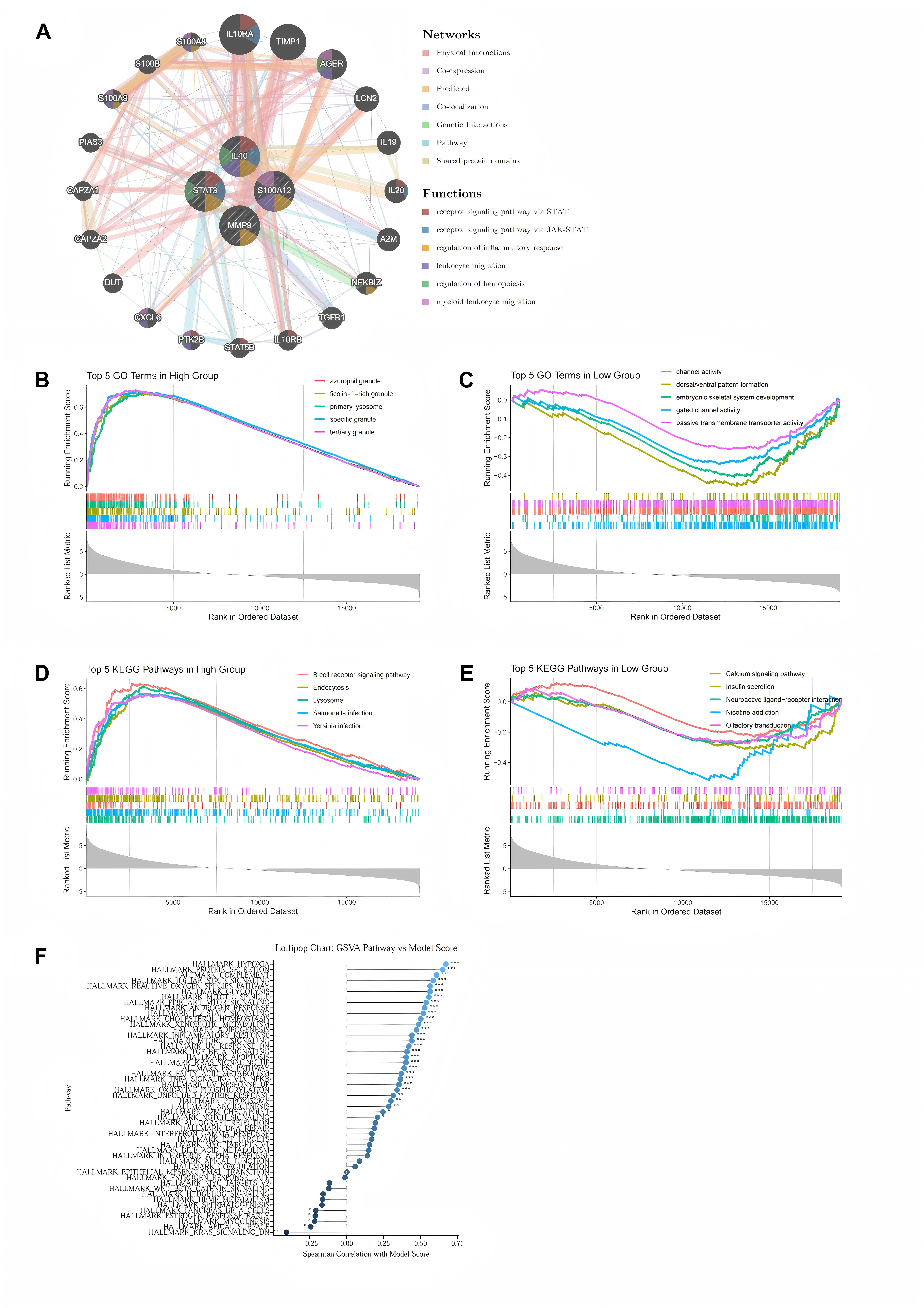

Supplement: Supplementary file 4 [file Image2.png]

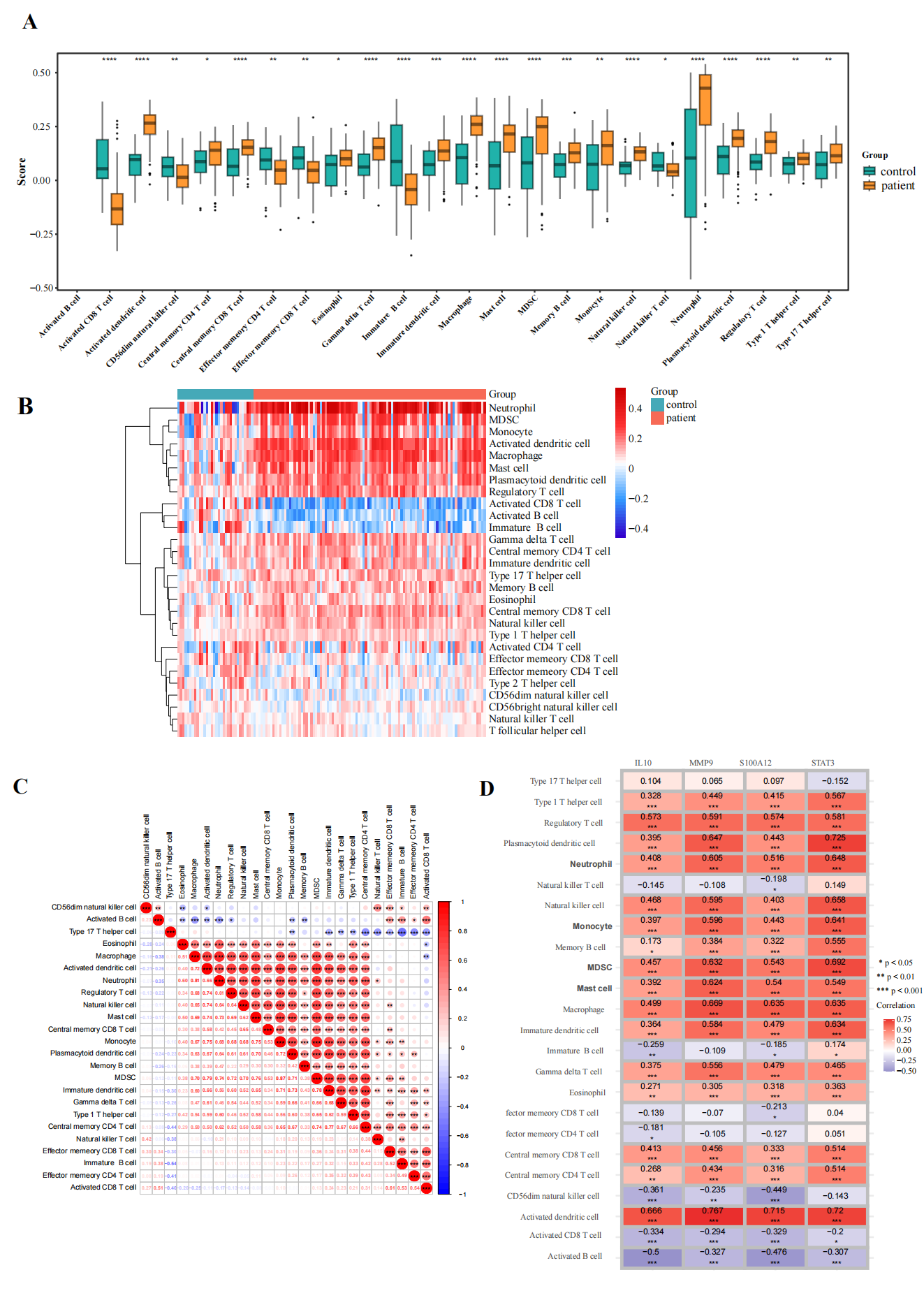

Supplement: Supplementary file 5 [file Image1.png]

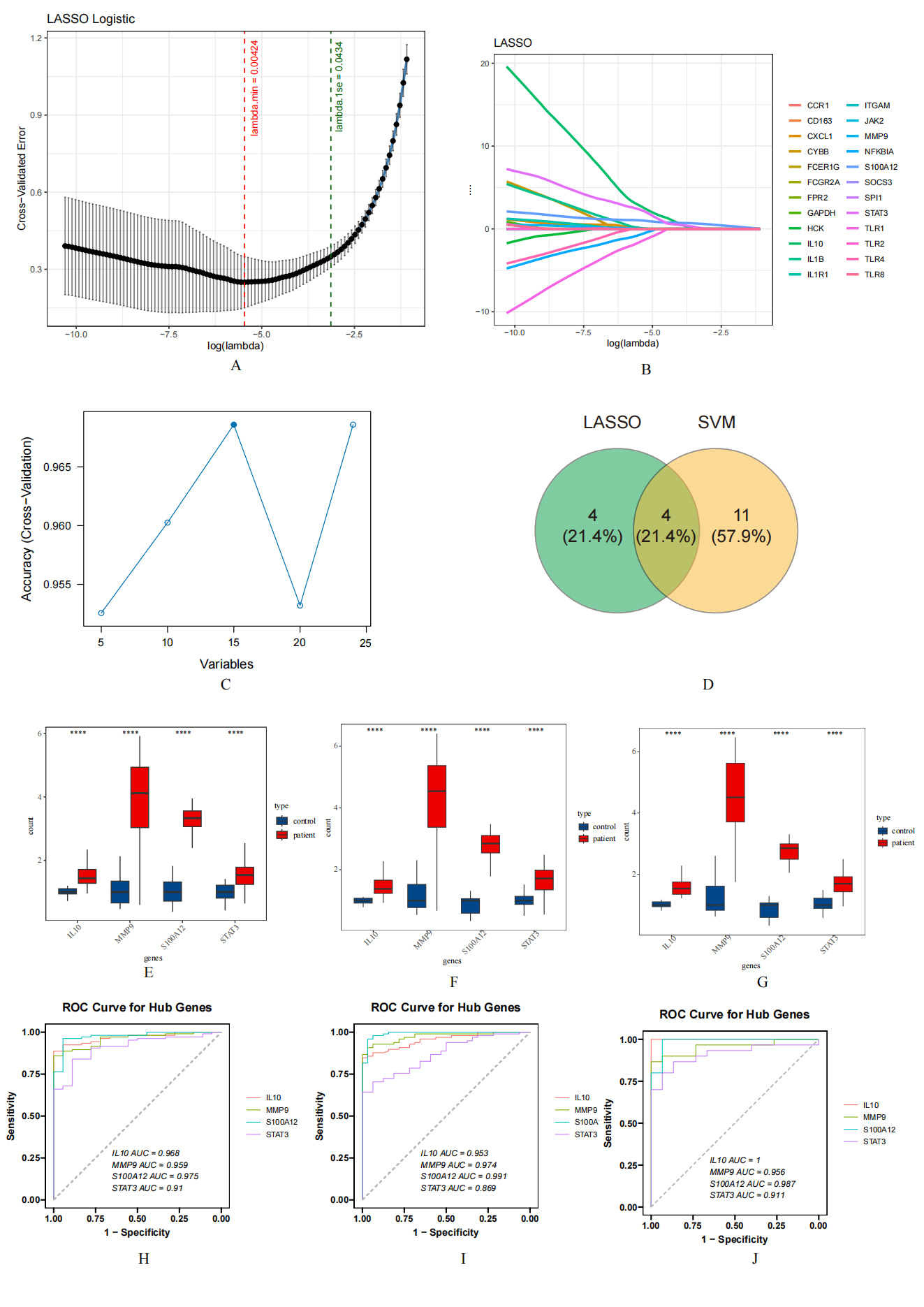

Supplement: Supplementary file 6 [file Image8.png]

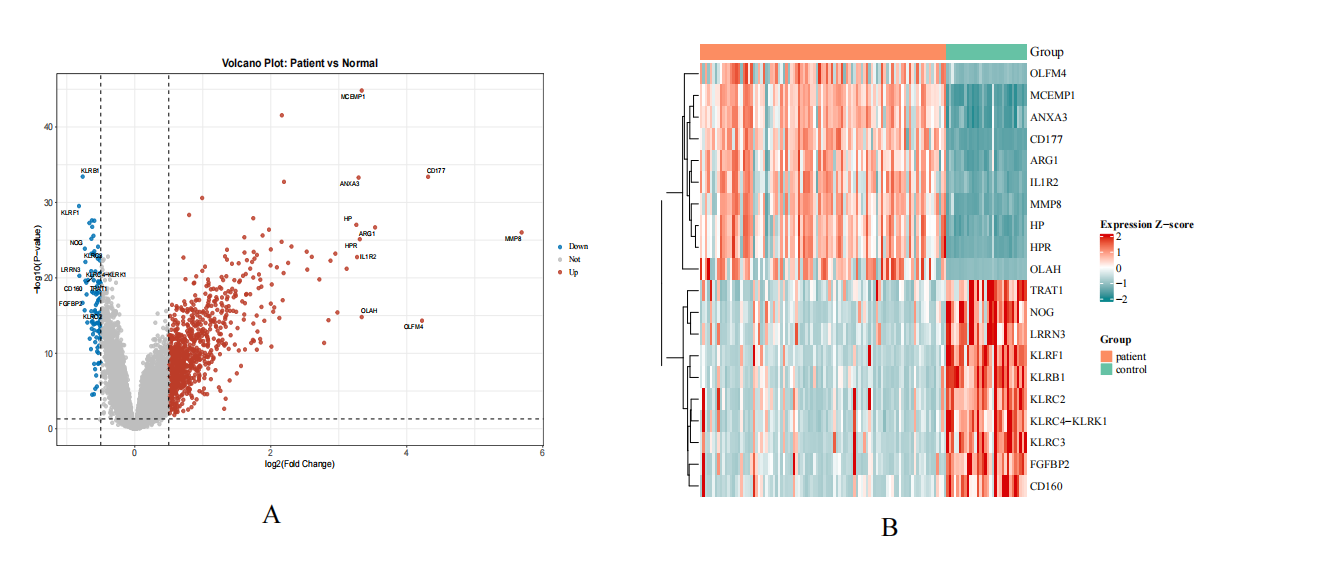

Supplement: Supplementary file 8 [file Image6.png]

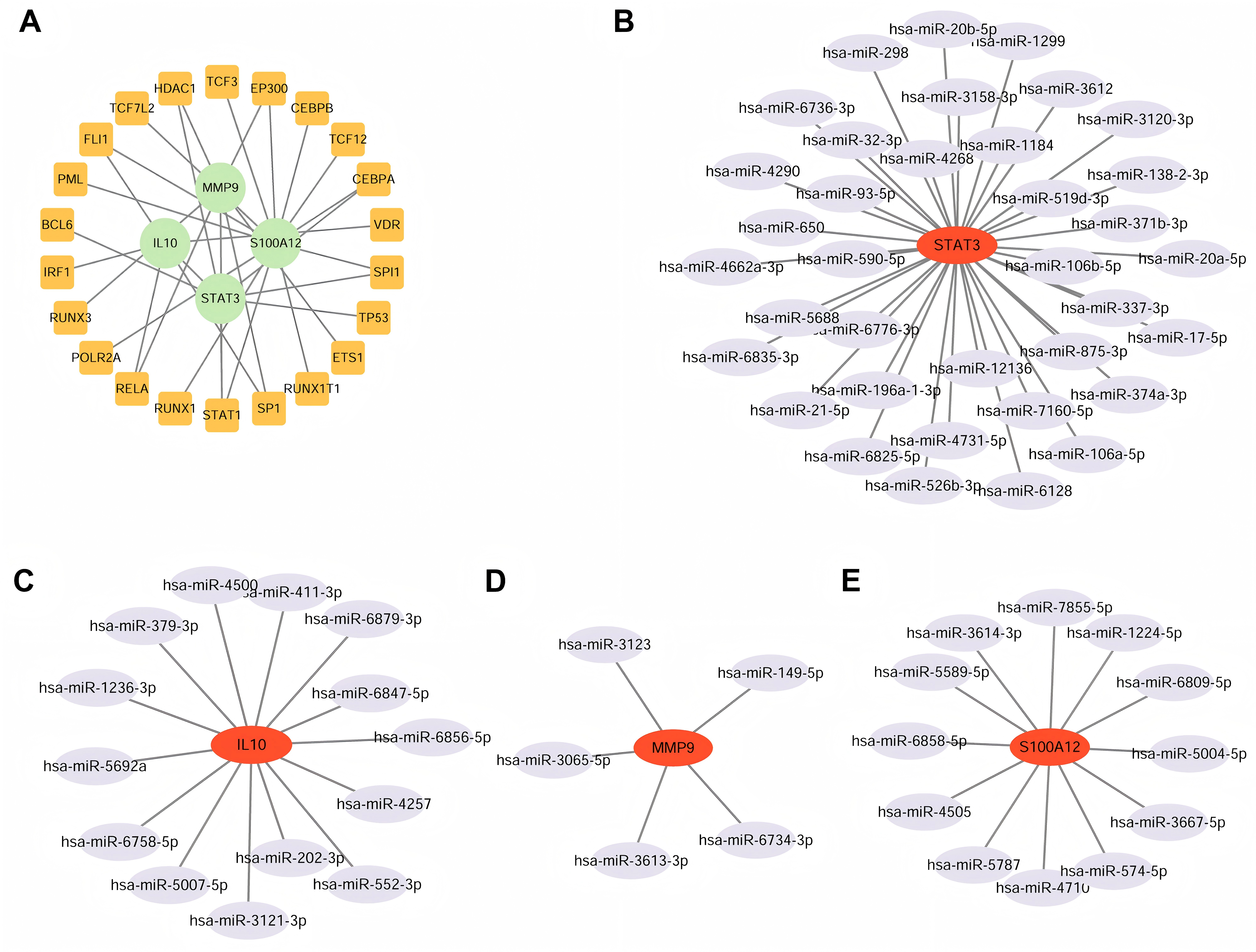

Supplement: Supplementary file 9 [file Image3.png]
